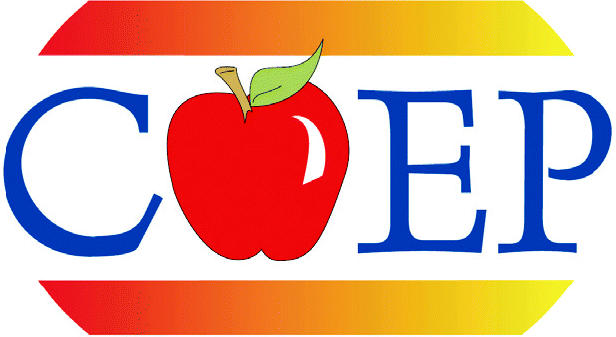# Beyond the Bench: Hunting Down Fugitive Literature

**Published:** 2004-10

**Authors:** Karalyn R. Colopy

The first step to becoming a successful fugitive is to abandon all forms of conventional identification—driver’s license, passport, checking account. People who stay out of the commercial realm are extremely hard to find. The same is true of literature. Libraries, with their online catalogs and helpful reference librarians, make it easy to find just about any piece of commercially published material. But lurking beyond the reach of the card catalog are thousands of materials such as reports, fact-sheets, newsletters, meeting transcripts, lesson plans, presentations, manuals, and interactive websites—so-called “fugitive literature”—that have never darkened the library’s door. Today, some of those fugitives have been found: the COEP Resource Center website (**http://www-apps.niehs.nih.gov/coeprc/**) offers visitors a bibliographic database for searching and reading about more than 600 environmental health materials developed by the Community Outreach and Education Programs, or COEPs, associated with each of the 25 NIEHS centers.

Since 1996, when the NIEHS established COEPs as an essential component of its Core Center Program, NIEHS grantees have been generating large volumes of fugitive literature. Charged with increasing public understanding of environmental health science research, the 25 COEPs carry out diverse projects. They host public forums and town meetings, offer professional development opportunities to teachers and health care providers, bring students to their laboratories for tours and summer science camps, and arrange for scientists to give presentations at local schools. They also develop curricula on environmental health for students in kindergarten through twelfth grade. These curricula are based on the latest research and are designed to meet state and national education standards.

The documents created during the course of the COEPs’ activities represent a wealth of environmental health information, innovative ideas, creative teaching approaches, lesson plans, videos, posters, brochures, training manuals, and successful outreach strategies. These materials are usually free and ready to use in a variety of education and outreach settings. However, until recently the people who could most use them—teachers, parents, nurses, community groups—were unlikely to find them.

That changed in 2000, when the NIEHS developed the COEP Resource Center to collect and catalog the products of the COEPs’ projects. Today, most printed resources are available for download in PDF format, and the database provides an abstract and ordering information for nonprint materials such as videos and CD-ROMs. The site also posts information about upcoming events, news, related links, and contact information for each COEP grantee.

The COEP Resource Center is now expanding its scope by incorporating materials produced by grantees in several other NIEHS programs besides the Core Center Program. Additions are planned over the next few months.

## Figures and Tables

**Figure:**